# *xbp-1* mRNA splicing is attenuated under prolonged exposure to ER stress

**DOI:** 10.17912/W2707X

**Published:** 2017-10-12

**Authors:** Jennifer Tsialikas, Yair Argon

**Affiliations:** 1 Department of Pathology and Laboratory Medicine, Children's Hospital of Philadelphia and University of Pennsylvania, Philadelphia, PA 19104

**Figure 1.**
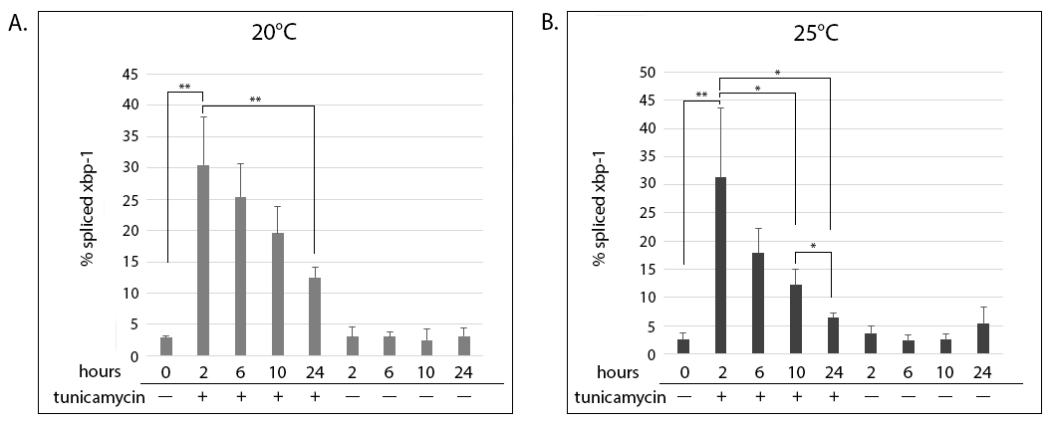


## Description

IRE-1 is an endoplasmic reticulum (ER) membrane-bound protein that mediates the unfolded protein response (UPR) when cells are under ER stress. Once activated, IRE-1 cleaves an intron from the *xbp-1* mRNA (Calfon *et al.*2002). This yields an active XBP-1 transcription factor, which activates transcription of ER chaperones and other genes involved in protein biosynthesis. Splicing of the *xbp-1* intron can be measured by a shift in size in an agarose gel (Shen *et al.* 2001). Previous studies in mammalian cells showed that there is a sharp increase in *Xbp-1* splicing following exposure to ER stress, but that this increase in splicing is attenuated under prolonged exposure to stress (Lin *et al.* 2007; Eletto *et al.* 2014). We asked whether this attenuation of *xbp-1* splicing is conserved in *C. elegans*.

Early L3 animals were put on tunicamycin containing plates (tunicamycin induces ER stress by blocking N-linked glycosylation (Dawson 1986)). They exhibited a sharp increase in *xbp-1* splicing after 2 hours of exposure (Figure 1A and B, compare 2 hours + tunicamycin to the 0-hour control). The maximal splicing seen at 2 hours decreased steadily for the duration of the time course at both temperatures. The attenuation of *xbp-1* splicing occurred faster in animals grown at 25°C (Figure 1B), where the reduction became statistically significant at 10 hours of exposure to tunicamycin (compare 2 and 10 hours + tunicamycin). In contrast, the reduction did not become significant in animals grown at 20°C until 24 hours of exposure (Figure 1A, compare 2 and 24 hours + tunicamycin). Control animals grown without tunicamycin did not exhibit spliced *xbp-1* above background levels throughout the time course (Figure 1A and B, – tunicamycin).

Tunicamycin treatment also affected the growth of the animals. At the 10-hour time point, when the majority of control animals had progressed to the early L4, tunicamycin-treated animals at both 20°C and 25°C were arrested at L3. Many animals had molting problems, like not exiting the old cuticle after shedding, or appearing to start ecdysis but bursting open during the process. At 24 hours, when the majority of control animals were young adults, tunicamycin-treated animals were still developmentally delayed. A considerable fraction were still at L3, and many animals had cuticle shedding abnormalities. Overall, there appeared to be more dead animals on the tunicamycin plates at 25°C compared to those grown at 20°C, but this was not quantified directly.

This time course showed that tunicamycin is a potent inducer of ER stress in *C. elegans* larvae, affecting molting and other aspects of developmental progression. Nonetheless, the UPR response of the population, as measured by *xbp-1* splicing, was still attenuated. Therefore, attenuation of the UPR stress response appears to be a conserved mechanism during chronic exposure to stress, not only in cultured mammalian cells, but also at the whole organism level. Given that a house-keeping gene was not assessed in parallel, a more widespread change that is not restricted to *xbp-1* cannot be excluded. Follow-up experiments should address the possibility that a more global alteration to RNA transcription and/or processing may be the cause of the attenuation. Furthermore, they should address whether the degree of stress, i.e. the concentration of tunicamycin, affects the attenuation response.

## Methods

A synchronous culture was obtained by bleaching gravid adults to isolate eggs, and allowing eggs to hatch overnight on an unseeded plate. Starved L1’s were collected with M9, moved to plates seeded with OP50, and placed at 20°C for 24 hours. Then, early L3 animals were handpicked onto plates which contained either 30mg/mL tunicamycin dissolved in DMSO, or an equivalent percentage of DMSO only, as a negative control. Plates were then incubated at either 20°C or 25°C. Fifty animals were collected manually at time 0 and after 2, 6, 10 and 24 hours, washed with M9, and frozen at -80°C in Trizol. RNA was extracted according to the manufacture’s protocol, and cDNA made using SuperScript II RT. 30ng of cDNA was amplified in each PCR reaction and resolved on 4% agarose gels. Percentage of spliced xbp-1 was calculated from the intensity of the bands using LiCor’s Image Studio Lite, as the percentage of signal from spliced xbp-1 divided by the total xbp-1 message and multiplied by 100. The graphs show the means of 3 biological replicates and standard deviations. Statistical significance was determined using Student’s *t-*test. * *P* £ 0.05, ** *P* £ 0.01.

## Reagents

Strain:SJ4005: zcIs4 [hsp-4::GFP; lin-15(n765)] V

Primers: Forward 5’ AGAAGTCGTCGGTGAGGTTG 3’ Reverse 5’ CGATCCATGTGGTTGCATAG 3’
